# *Porphyromonas gingivalis* Infection Reduces Regulatory T Cells in Infected Atherosclerosis Patients

**DOI:** 10.1371/journal.pone.0086599

**Published:** 2014-01-23

**Authors:** Jie Yang, Juan Wu, Yu Liu, Jin Huang, Zhipin Lu, Liping Xie, Weibin Sun, Yong Ji

**Affiliations:** 1 Department of Periodontology, Hospital of Stomatology, Medical School, Nanjing University, Nanjing, Jiangsu Province, China; 2 Department of Cardiology, Nanjing Chest Hospital, Nanjing, Jiangsu Province, China; 3 Key Laboratory of Cardiovascular Disease and Molecular Intervention, Atherosclerosis Research Centre, Nanjing Medical University, Nanjing, Jiangsu Province, China; 4 Central Laboratory of Stomatology, Hospital of Stomatology, Medical School, Nanjing University, Nanjing, Jiangsu Province, China; The University of Texas Medical Schoo at Houstonl, United States of America

## Abstract

Increasing evidence has shown periodontal pathogen *Porphyromonas gingivalis (P.gingivalis)* infection contributes to atherosclerosis (AS) progression. *P.gingivalis* fimbriae act as an important virulence factor in AS. Regulatory T cells (Tregs) may play a crucial role in autoimmune response during this process. However, whether *P.gingivalis* infection is associated with Tregs dysregulation during AS is still unknown and the prevalence of different *P.gingivalis* FimA genotypes during this process is unclear. Here we analyzed the distribution of Tregs and in *P.gingivalis*-infected atherosclerotic patients to reveal the relationship between *P.gingivalis* infection and Tregs reduction/dysfunction and to elucidate their role in periodontitis-AS interaction. FimA genotype was also examined to determine the prevalence of fimbriae. Our results showed that *P.gingivalis* infection reduced Tregs in atherosclerotic patients compared with non-atherosclerotic patients and health controls. Concentration of TGF-β1, which plays an important role in the development of Tregs, also decreased in *P.gingivalis* infected patients. Furthermore, type II FimA seems to show higher prevalence than the other five detected types. The population of Tregs further decreased in patients with type II FimA compared with the other types. *P.gingivlias* FimA genotype II was the dominant type associated with decreased Treg population. These results indicate that *P.gingivalis* infection may be associated with Tregs dysregulation in AS; type II FimA may be a predominant genotype in this process.

## Introduction

Atherosclerosis is one of the most common causes of death in many countries [1]. Risk factors such as hypertension, high cholesterol and smoking were thought to be related with AS, however, observation discovered that half of the patients suffered from AS lack these risk factors [2], [3]. More and more evidence support the contention that AS is an inflammatory disease, host immune response plays an important role in the pathogenesis of AS [4], [5].

Chronic periodontitis is an inflammatory disease in periodontal tissue resulted from oral infection of periodontal pathogens. Accumulating evidence indicated a close relationship between periodontal infection and AS [6]–[8]. *P.gingivalis*, as the main pathogen of chronic periodontal disease, is a gram-negative anaerobic bacterium in the subgingival dental plaque. Encoding *P.gingivalis* fimbrillin, FimA gene could be classified into six genotypes based on the DNA sequence. Strains expressing different genotypes of FimA exhibit various pathogenicities in the progress of periodontitis [9]. As bacterial infection is the initial etiology for periodontal disease, local severe inflammation can lead to gingival ulceration and epithelial barrier destruction, which increases the incidence of *P.gingivalis* translocation into circulation system. Clinical studies have detected *P.gingivalis* in serum or plaque of AS patients [10]. Our previous experiments also demonstrated *P.gingivalis* can invade endothelial cells and promote endothelial dysfunction [11]. Molecular mimicry between bacterial antigenic peptides and mammalian protein will lead to the autoimmune responses, which is an important mechanism of periodontal infection-associated AS [12]. *P.gingivalis* can induce cross-reaction against endothelial cells via Heat Shock Protein 60 (HSP60), and the reaction to HSP60 in endothelial cells will finally activate CD4^+^ T cells mediated-autoimmune response [13], [14]. Moreover, recent research indicated that there was a close relationship between *P.gingivalis* infection and the accumulation of CD4^+^ T cells in periodontal lesions [15]. In all, *P.gingivalis* infection may participate in AS by inducing CD4^+^ T cell response.

T cells play a central role in cellular immunity. There are several subsets such as T helper cells, cytotoxic T cells and regulatory T cells, each with a distinct function. Tregs play crucial roles in maintaining immune system homeostasis. Tregs suppress CD4^+^ and CD8^+^ effector T cells immune responses, thereby modulating adaptive immune responses, and maintaining self-tolerance [16]. Cytokines such as IL-10 and TGF-β1 are produced by Tregs and are implicated in Tregs function. It has been demonstrated Tregs are efficient in the control of autoimmunity [17]. Importantly, Tregs act as inhibitors of AS [18], [19]. Upregulation and transfer of Tregs can inhibit the induction of T cells and macrophages into plaque. Several independent studies showed that Tregs produce high levels of IL-10 and lead to a decrease in the process of atherosclerotic plaques formation [20], [21]. Increase of Tregs can promote the stability of AS plaque, while depletion of Tregs promotes hypercholesterolemia and AS [22]. However, it is still largely unknown if Tregs mediate the interaction between periodontitis and AS. The potential role of *P.gingivalis*, which represents dominant pathogen in periodontitis, in immune system dysregulation during AS also remains unclear.

Therefore, in this study, we examined the level of Tregs in peripheral blood of *P.gingivalis* infected atherosclerotic patients to analyze the relationship between *P.gingivalis* infection and Tregs distribution and to elucidate their role in periodontitis-AS interaction. Furthermore, we studied the prevalence of different *P.gingivalis* strains in the process.

## Materials and Methods

This study was approved by the Ethics Committee of Hospital of Stomatology, Medical School, Nanjing University (NF2012-021), and conducted according to the standards of the Declaration of Helsinki. Informed written consent was obtained from all participants.

### Human Subjects

The study was approved by the ethics committees of Hospital of Stomatology, Medical School, Nanjing University. Informed written consent was obtained from all subjects. All the patients and volunteers were examined carefully on periodontal parameters such as oral hygiene, number of teeth loss and oral panoramic radiograph to diagnose periodontitis. Electrocardiography, echo-cardiograms and carotid artery ultrasonography were employed to determine atherosclerosis risk.

Among these subjects, a total of 40 patients were diagnosed as atherosclerosis with significant stenosis (>50%) on angiography as well as moderate to severe periodontitis after careful oral examination in Nanjing Chest Hospital (Pg-AS patients). 32 patients with teeth loss and alveolar bone absorption but with neither clinical symptoms of atherosclerosis risk factors, nor proof to diagnosis as atherosclerosis by carotid artery sonography (less than 9mm in carotid artery intima-media thickness), normal electrocardiography and echocardiograms were diagnosed as periodontitis only (Pg patients). 29 subjects with no clinical symptoms of AS, no risk factors and no periodontal infection were included as health controls (HCs). The electrocardiography, echo-cardiograms and carotid artery sonography examination were all normal.

Exclusion criteria included patients with malignancy, immunodeficiency disease, diabetes, patients on immunosuppressive drugs, or patients underwent infection in the last 3 months. All study subjects need undergo a clinical examination thoroughly. A history of past and current smoking was contained. All clinical features and laboratory investigation were recorded in detail.

### IgG Titers Against *P.gingivalis* Measurement

The titers of *P.gingivalis* specific plasma IgG antibody of subjects were tested by enzyme-linked immunosorbent assay (ELISA) [23]. Sonicated preparations of *P.gingivalis* ATCC33277 were used as bacterial antigens. To adjust the analyses, plasma from 5 healthy volunteers without periodontitis and AS were chosen to pool. Standard titration curves were prepared by diluting the control plasma. All ELISA data were expressed in ELISA unit (EU), derived by relating OD_405_ values from each test serum to the corresponding reference serum. 100 EU was defined as correspond to 1:3,200 dilution of the sample.

### Flow Cytometric Analysis

Heparinized blood was obtained from each subject. Ficoll-Hypaque density gradient centrifugation (TBD Sciences, Tianjin, China) was used to isolate peripheral blood mononuclear cells (PBMCs). After isolation, PBMCs were incubated with a cocktail of two fluorescent monoclonal antibodies (BD Biosciences, San Jose, CA) directed to human CD25 (allophycocyanin), and CD4 (fluorescein isothiocyanate) for surface marker analyses for 30 minutes at 4°C. After washing with PBS, cells were treated with fixation permeabilization reagents (no. 560098, BD Biosciences) or the FOXP3/Transcription Factor Fixation/Permeabilization buffer (no. 00-5521, eBioscience), then incubated with anti-human FOXP3 antibody clone 259D/C7 (BD Biosciences, San Jose, CA), or clone 236A/E7 (eBioscience, San Jose, CA) for 30 minutes at 4°C to stain intracellular marker. Isotype antibodies were also used. All samples were detected on a FACSCalibur flow cytometer (BD Biosciences). A minimum 10,000 cells in the lymphocyte gate (forward scatter/side scatter) were acquired and analyzed with FlowJo software (BD Biosciences). Cell number was determined by multiplying the total cell count by frequency.

### TGF-β1 ELISA Assay

Fresh heparinized blood samples were centrifuged to obtain plasma. All samples were stored at − 80°C after centrifugation immediately. Plasma cytokine transforming growth factor-beta1 (TGF-β1) concentration was measured by ELISA with ELISA kit (eBioscience, USA) following manufacturer’s instruction.

### Subgingival Plaque

Periodontal examination was performed carefully. The number of teeth was recorded. Subgingival plaque samples were then processed from the deepest periodontal sites with periodontal depth ≥5 mm with sterile Gracycurettes. Bacterial genomic DNA was isolated from these samples with MiniBEST Bacterial Genomic DNA Extraction Kit (Takara). The isolated DNA was stored at −20°C. *P.gingivalis* fimA genotype was determined by PCR method. To analyze fimA gene, the specific primers for each subtype described by Hayashi et al [24] were used. All PCR products were viewed by electrophoresis. In each sample processing, we set controls to avoid false positives and negatives.

### Statistical Analysis

All data was analyzed by the Shapiro–Wilk test to determine normal distribution. CD4^+^CD25^+^FOXP3^+^Tregs frequencies, cell numbers, TGF-β1 concentration and *P.gingivalis* antigen EU values were abnormal distribution. Values among three groups were compared by the Kruskal–Wallis H test. Every two groups were tested by the Mann-Whitney *U* test, after Bofferoni correction, a value of *P*<0.0167 was considered as significant difference. Values following a normal distribution were compared by Mann-Whitney *U* test among two groups, a value of p<0.05 was considered as significant different. All statistical analysis was performed with SPSS18.0.

## Results

### Patients Characteristics

Age, gender and frequency of smoking showed no difference in patients with Pg-AS, Pg and HC groups. Patients with Pg-AS had more teeth loss than Pg patients and controls. To evaluate the immune reaction to *P.gingivalis* infection, the levels of IgG antibody to *P.gingivalis* were measured by ELISA. The results showed that antibody titers in Pg group 224EU (122-528EU) and Pg-AS group 327EU (206-871EU) significantly elevated when compared with that of normal subjects 95EU (48-131EU). Furthermore, the Pg-AS group’s antibody level was even higher than Pg group (*P*<0.0167) (Table 1).

**Table 1:** Demographic characteristics and clinical parameter of the study population.

	HC	Pg	Pg-AS
Number of cases	29	32	40
Age(yrs)	59.2±6.23	61.7±9.1	66.2±8.5
Gender(% Male)	44.8	53.1	57.5
Smokers (%)	27.5	34.3	30
Loss of teeth	0	5.26±0.89*	8.34±1.39*^,#^
*P.gingivalis* IgG Abtiter(EU)	95 (48–131)	224 (122–528)*	327 (206–871)*^,#^

Continuous variables expressed as mean ± standard deviation, percentage or number. *P<0.01 Pg and Pg-AS vs. control; ^#^ *P*<0.01 Pg-AS vs. Pg. HC = health control; Pg = *P.gingivalis* infected patients; Pg-AS = *P.gingivalis* infected atherosclerosis patients.

### Analysis of CD4^+^CD25^+^FOXP3^+^Tregs Level in Peripheral Blood

**Figure 1:**
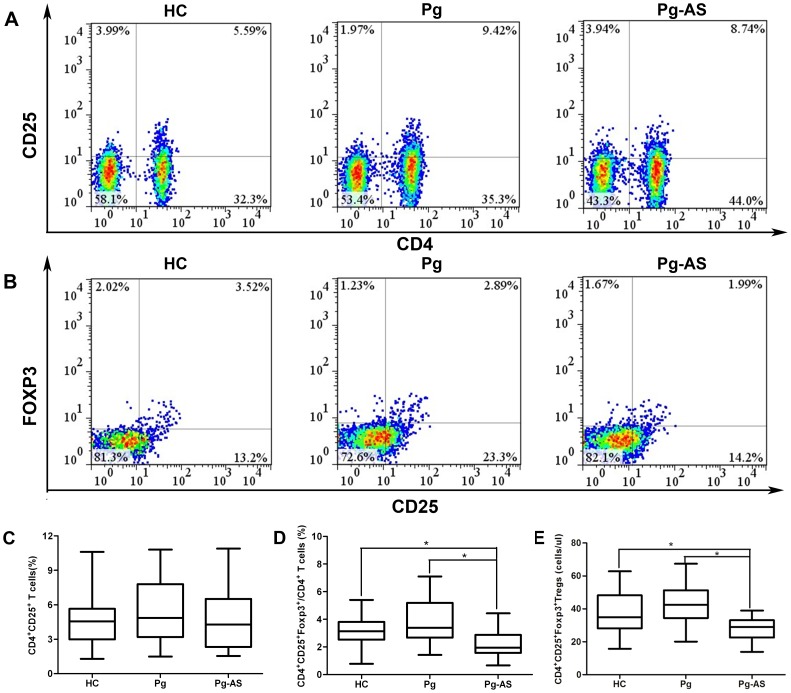
The percentage and absolute number of CD4^+^CD25^+^ T cells and CD4^+^CD25^+^FOXP3^+^ T cells in Pg-AS patients, Pg patients, and HCs. A. Representative profiles of Pg-AS, Pg and HC for detecting CD4^+^CD25^+^ T cells by flow cytometry. A minimum 10,000 cells in the lymphocytes gate were acquired. B. Representative profiles of Pg-AS, Pg and HC for detecting CD4^+^CD25^+^FOXP3^+^ T cells by flow cytometry within the CD4^+^ gate. C. The percentage of CD4^+^CD25^+^ T cells in Pg-AS (n = 40), Pg (n = 32) and HC (n = 29) groups. D. The percentage of CD4^+^CD25^+^FOXP3^+^ T cells in CD4^+^ T cells in Pg-AS (n = 40), Pg (n = 32) and HC (n = 29) groups. CD4^+^CD25^+^ T cells and CD4^+^CD25^+^FOXP3^+^ T cells frequency were shown individually. E. CD4^+^CD25^+^FOXP3^+^ T cells number (per ul) was calculated in Pg-AS (n = 40), Pg (n = 32), HC (n = 29) groups. Boxes with the 25th to 75th percentiles and the lines with the 5th to 95th percentiles are presented.*P<0.0167. HC = health control; Pg = *P.gingivalis* infected patients; Pg-AS = *P.gingivalis* infected atherosclerosis patients.

PMBC from Pg-As patients, Pg-infected patients, and HC donors were obtained. The percentage of CD4^+^CD25^+^ T cells (Figure 1A) in PBMC and CD4^+^CD25^+^FOXP3^+^ Tregs in total CD4^+^ T cell (Figure 1B) was determined by flow cytometry. In Pg-As patients, the CD4^+^CD25^+^ T cells represented 1.55% to 10.95% of CD4^+^ T cells; the CD4^+^CD25^+^FOXP3^+^ Tregs population varied from 0.67% to 4.43%. In Pg-infected patients, CD4^+^CD25^+^ T cells frequency represented from 1.49% to 10.80%, the percentage CD4^+^CD25^+^FOXP3^+^ Tregs comprised 1.43% to 7.10%. In HCs, CD4^+^CD25^+^ T cells population varied from 1.30% to 10.60%, and CD4^+^CD25^+^FOXP3^+^ Tregs represented 0.78% to 5.40%. As a result, the frequencies of CD4^+^CD25^+^ T cells in Pg-AS group were 4.27% (1.55–10.95%), in Pg group and HC group were 4.85% (1.49–10.80%) and 4.56% (1.30–10.60%) respectively. No significant difference were found in peripheral CD4^+^CD25^+^ T cells among these three groups (*P*>0.05) (Figure 1C). However, the frequencies of CD4^+^CD25^+^FOXP3^+^ Treg cells in CD4^+^T cells in Pg-AS patients were 1.95% (0.67–4.43%), they were significantly lower than that in Pg-infected patients (median 3.38% range 1.43–7.10%) (*P*<0.0167) and controls (median 3.14% range 0.78–5.40%) (*P*<0.0167). Statistical analysis results showed no significant difference in the population of CD4^+^CD25^+^FOXP3^+^/CD4^+^ T cells between Pg-infected group and HC group (Figure 1D). Furthermore, the absolute number of Tregs in the peripheral blood was calculated. In patients with Pg-AS, the number of CD4^+^CD25^+^FOXP3^+^ Tregs was 29 cells/ul (13–39) cells/ul, it was lower than that in HC (median 36 cell/ul range 17–63 cells/ul) and Pg group (median 42 cell/ul range 20–66 cells/ul)(P<0.0167) (Figure 1E). While no significant difference was found between Pg and HC groups.

**Figure 2:**
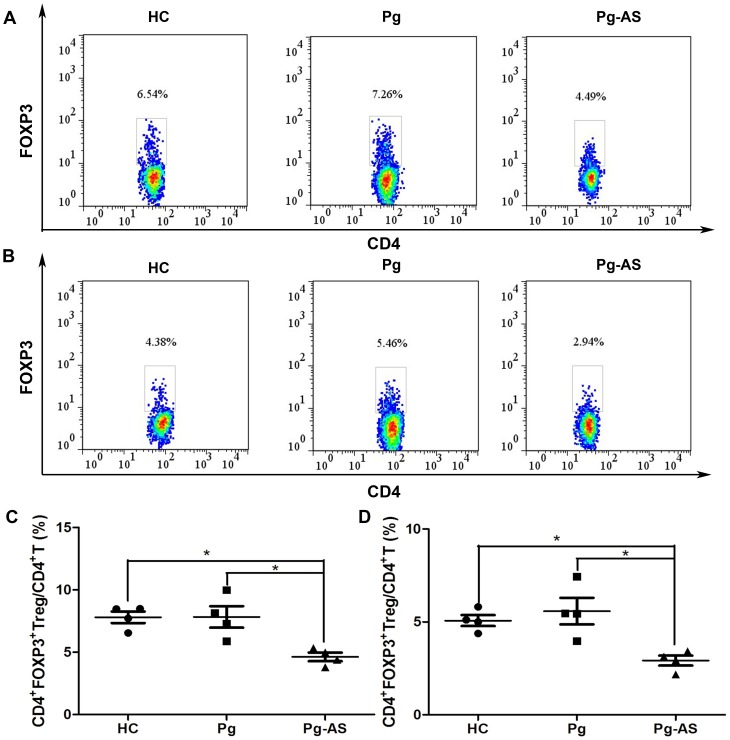
The percentage of CD4^+^FOXP3^+^ T cells in Pg-AS patients, Pg patients, and HCs. A. Representative profiles of Pg-AS, Pg and HC for detecting CD4^+^FOXP3^+^ T cells by flow cytometry within the CD4^+^ gate. Cells were stained with anti-FOXP3 monoclonal antibody (clone 236A/E7) and Fixation/Permeabilization buffer (no. 00-5521) from eBioscience. B. Representative profiles of Pg-AS, Pg and HC for detecting CD4^+^FOXP3^+^ T cells by flow cytometry within the CD4^+^ gate. Cells were stained with anti-FOXP3 monoclonal antibody (clone 259D/C7) and fixation permeabilization reagents (no. 560098) from BD Bioscience. C. The percentage of CD4^+^FOXP3^+^ T cells stained with eBioscience reagents in Pg-AS (n = 4), Pg (n = 4) and HC (n = 4) groups. D. The percentage of CD4^+^FOXP3^+^ T cells stained with BD reagents in Pg-AS (n = 4), Pg (n = 4) and HC (n = 4) groups. Boxes with the 25th to 75th percentiles and the lines with the 5th to 95th percentiles are presented.*P<0.0167. HC = health control; Pg = *P.gingivalis* infected patients; Pg-AS = *P.gingivalis* infected atherosclerosis patients.

As staining condition such as the antibody source and staining buffer combination would affect the results, four donors in each group were selected randomly to staining with two anti-FOXP3-PE antibodies from different sources (clone 236A/E7 from eBioscience and 259D/C7 from BD Biosciences). The percentage of CD4^+^FOXP3^+^ Tregs in CD4^+^ T cells was determined (Figure 2A and 2B). Even though the number tested with 236A/E7 was higher than that with 259D/C7, the tendency was similar and stably indicated that the percentage of CD4^+^FOXP3^+^ Tregs in CD4^+^ T cells decreased in patients with Pg-AS compared with HC and Pg (P<0.0167) (Figure 2C and 2D).

### Plasma TGF-β1 Level

**Figure 3:**
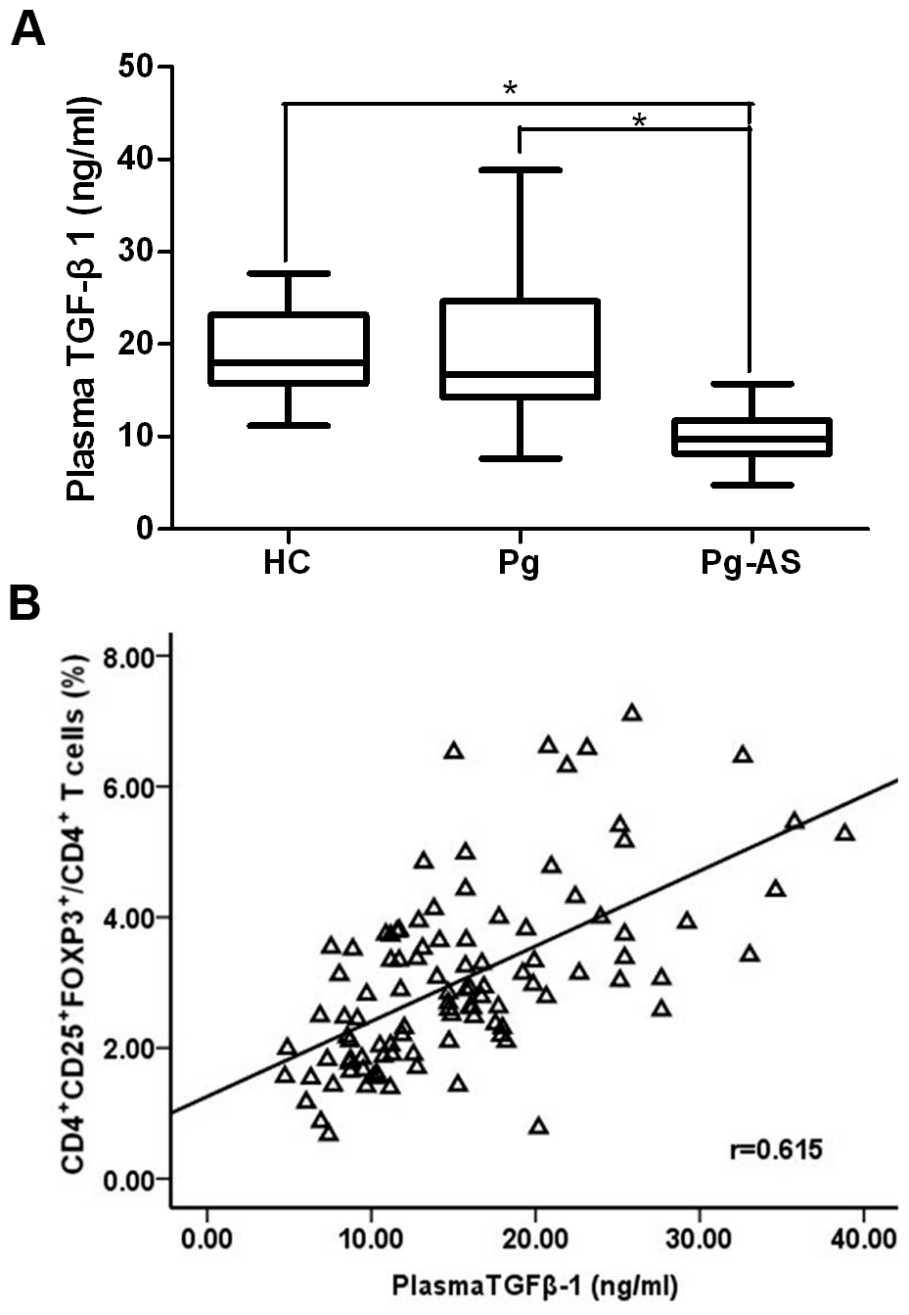
Plasma TGF-β1 concentration in Pg-AS patients, Pg patients, and HCs. A. Plasma TGF-β1 concentrations in HC (n = 29), Pg (n = 32), Pg-AS (n = 40) groups were presented. Boxes with the 25th to 75th percentiles and the lines with the 5th to 95th percentiles are shown.*P<0.0167. B. Spearman correlation between frequencies of circulating TGF-β1 and CD4^+^CD25^+^FOXP3^+^/CD4^+^ T cells in these groups (n = 101). HC = health control; Pg = *P.gingivalis* infected patients; Pg-AS = *P.gingivalis* infected atherosclerosis patients.

Previous studies showed that TGF-β1 level was associated with atherosclerotic plaque formation. We also determined the cytokine TGF-β1 level by ELISA. A decreased level of TGF-β1 was found in Pg-AS patients (median 9.69 ng/ml range 4.73–15.73 ng/ml) compared with those in Pg patients (median 16.71 ng/ml range 7.67–38.84 ng/ml) and HC (median 17.97 ng/ml range 11.17–27.66 ng/ml) (Figure 3A, all *P*<0.0167). A strong correlation between the frequency of CD4^+^CD25^+^FOXP3^+^/CD4^+^ T cells and plasma TGF-β1 concentration was detected (r = 0.615, *P*<0.001) (Figure 3B).

### Distribution of Six FimA Types in Subgingival Plaque of Patients Carrying *P.gingivalis*

**Figure 4:**
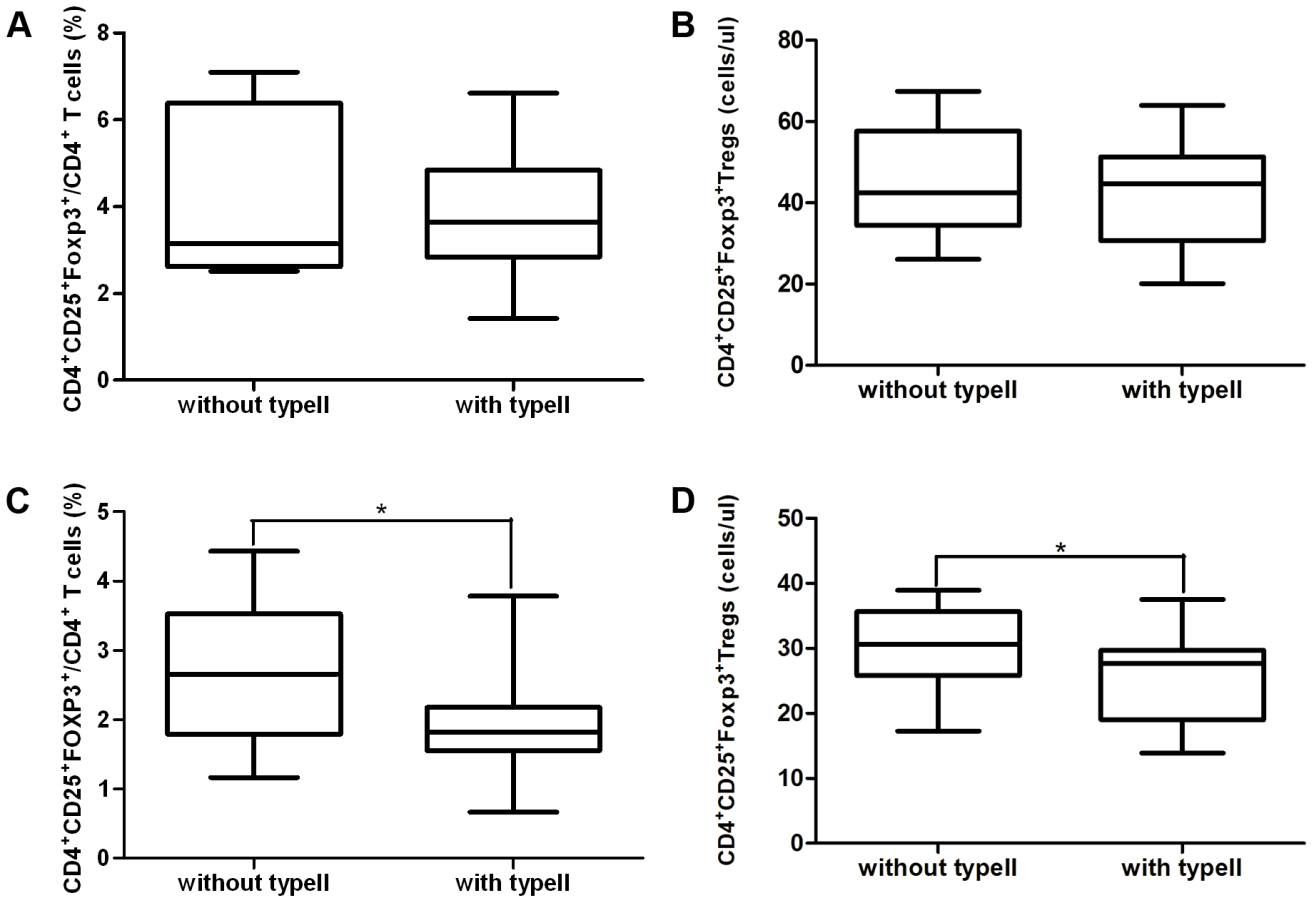
Genotype II infection and circulating CD4^+^CD25^+^ FOXP3^+^/CD4^+^ T cells. A and B. The frequency and cell number of CD4^+^CD25^+^FOXP3^+^/CD4^+^ T cells comparison in Pg patients between with type II FimA (n = 19) and without type II FimA (n = 13) *P.gingivalis* in the subgingival plaque. C and D. The frequency and cell number of CD4^+^CD25^+^FOXP3^+^/CD4^+^ T cells comparison in Pg-AS patients between with type II FimA (n = 26) and without type II FimA (n = 14) *P.gingivalis* in the subgingival plaque. *P<0.05.

Table 2 shows the distribution of different FimA genotypes found in the subgingival plaque of Pg and Pg-AS patients. Genotype II (59.4% and 65%) was present in a significantly higher percentage than the other genotypes in both Pg and Pg-AS patients. As type II is the dominant genotype of *P.gingivalis*, we compared the frequency of CD4^+^CD25^+^FOXP3^+^/CD4^+^ T cells in patients infected with genotype II with those without genotype II. There was no difference between patients with type II and without type II in Pg group (Figure 4A and 4B). The frequency of CD4^+^CD25^+^FOXP3^+^ T cells was significantly decreased in genotype II *P.gingivalis* infected AS patients [without type II *vs.* with type II 2.65% (1.17–4.43%) *vs.* 1.82% (0.67–3.78%)] (*P*<0.05) (Figure 4C and 4D). Similarly, the absolute Treg cell number also decreased in AS patients with type II *P.gingivalis* infection [without type II *vs.* with type II 30 cells/ul (17–39 cells/ul) *vs.* 27 cells/ul (13–37 cells/ul)] (*P*<0.05).

**Table 2:** Distribution of 6 FimA types in Pg-AS and Pg patients.

FimA genotype	Frequency of occurrence (%)	
	group of subjects	
	Pg	Pg-AS
I FimA	31.25	22.5
Ib FimA	31.25	27.5
II FimA	59.4	65
III FimA	15.6	17.5
IV FimA	37	32.5
V FimA	0	2.5

Pg = *P.gingivalis* infected patients; Pg-AS = *P.gingivalis* infected atherosclerosis patients.

## Discussion

Both innate immunity and acquired immunity participate in the atherogenic process [4]. Immune cells, particularly monocytes and T lymphocytes, are implicated in the progress. Hansson *et al*. discovered T cells in atherosclerotic plaques and these cells made a contribution to the progression of inflammatory condition [5]. T lymphocytes can be classified into different subsets with different functions. In the progression of AS, Tregs can inhibit the rupture of AS plaque. On the other hand, T helper cells such as Th1 cells, accelerate the development of AS plaque formation by secreting cytokines as IL-2, IFN-r and TNF- α [25], [26]. Th2 cells produce and secrete cytokines such as IL-4, IL-5, and IL-10 to help B cells to produce antibody. The activity of these effector T cells is regulated by Tregs strictly [27]. Tregs produce large amounts of TGF-β and IL-10 and play an important role in the process of atherosclerosis by repressing immune function and thus provide a promising target for the modulation of the disease. Tregs reduction or dysfunction are important in the etiology of AS [19], [20], [22]. In recent years, accumulating amount of studies support an association between *P.gingivalis* and AS. *P.gingivalis* infection promotes the expression of cytokines, chemokines or adhesion molecular such as IL-8, MCP-1, ICAM-1 and VCAM-1 in endothelial cells, as well as the adhesion of monocyte to vascular endothelial, which may contribute to atheroma formation [28], [29]. However, studies on the association between *P.gingivalis* infection and Tregs in the progression and development of AS are relatively insufficient. Considering that the balance between pro- and anti-inflammatory is a major determination of disease progression, we detected the immune reaction to *P.gingivalis* infection and analyzed Tregs distribution in atherosclerotic patients.

CD4^+^CD25^+^ Tregs, as a new subset of T cells, act as a central in modulating immune system and maintaining tolerance self-antigens. FOXP3, as a good marker for CD4^+^CD25^+^ Tregs, is found to confer suppressive function on peripheral CD4^+^CD25^+^ Tregs [30]. Studies in both humans and animal models have demonstrated that decrease of Tregs in peripheral blood contributes to immune disorder related diseases [31], [32]. Therefore, Tregs may play a crucial role during AS development [20]. We found that the population of CD4^+^CD25^+^ FOXP3^+^/CD4^+^ T cells decreased in patients with AS although CD4^+^CD25^+^ T cells remain unchanged. According to the fact that immune response to *P.gingivalis* in atherosclerotic patients is stronger than periodontitis patients, we believe the decrease of CD4^+^CD25^+^FOXP3^+^ T cells be related with the increased response towards *P.gingivalis* infection. The sustained IL-2 level is important for the growth of Tregs. *P.gingivalis* can suppress the accumulation of IL-2 and attenuate T cells proliferation to alter adaptive immune responses in the process of atherosclerosis [33]. Moreover, TGF-β1 is one of the cytokines secreted by Tregs and is associated with the survival and function of Tregs [34]. TGF-β1 plays a crucial role in promoting Tregs differentiation by regulating the signaling pathway [35]. In this study, the level of TGF-β1 in peripheral blood of Pg-AS patients decreased. The decreased TGF-β1, as well as the close relationship between TGF-β1 concentration and Tregs frequencies implied that *P.gingivalis* may impair the production of TGF-β1 to inhibit Tregs differentiation which may promote pro-atherogenic responses. The decrease of Tregs population in peripheral blood may result from proliferation inhibition or differentiation impairment. Furthermore, inflamed gingival tissue was reported to contain a high frequency of FOXP3^+^ Treg cells [36], [37], which indicated that local inflammation in gingival tissue may recruit Tregs and subsequently lead to the reduction of circulating Tregs in peripheral blood.

*P.gingivalis*, as a gram-negative anaerobic bacterium, can produce various virulence factors such as lipopolysaccharides, gingipain and fimbriae. FimA fimbriae play vital roles in bacterial colonization and invasion. According to the nucleotide sequences, *P.gingivalis* FimA can be classified into six variants (types I∼V and Ib) [24]. Different genotypes possess different virulence capabilities. Fimbriae expression and different fimbriae types showed significant difference in pro-atherogenic effects in previous studies [7], [38], [39]. Our study showed genotype II, in subgingival plaque of the AS patients, was more prevalent than the other types. This result is consistent with previous studies reporting that fimA genotype II was associated with more aggressive forms of diseases [38], [40]. In the development of AS, type II *P.gingivalis* can conjugate to form microspheres and invade human epithelial cells most efficiently among the six types. Infection of type II *P.gingivalis* also exhibited prolonged cytokine response such as IL-1b, IL-8 and TNFα [41], [42]. Our results demonstrated that the decreased percentage and number of Tregs may be related with genotype II infection. Patients infected with this genotype may have a higher risk of atherosclerosis.

Patients with poor oral health were more possibly to get myocardial infarction and cerebrovascular disease in a multiple regression analysis. Oral hygiene was an independent factor except for age, cholesterol or hypertension [43]. Dental status, particularly the number of tooth loss is closely associated with the degree of carotid stenosis [44]. Although oral clinical signs show association with AS, they do not reflect systemic reaction activated by the periodontal pathogen. Microbiologic aspects and infectious markers of periodontal disease are more specific than clinical parameters of periodontitis [45], [46]. The systemic antibody response to *P.gingivalis* has a positive correlation with the spread of the pathogen in peripheral blood [47]. The systemic reaction to *P.gingivalis* reflects the carriage of the periodontal pathogen for patients. The quantity of bacterial exposure, rather than clinical measures, is more important to systemic health [48]. Therefore it’s more relevant to detect immune reaction index to display pathogen infection. In this study, *P.gingivalis* infection was determined by measuring the specific IgG titer in peripheral blood. Our study found atherosclerotic patients of *P.gingivalis* infection showed more teeth loss and higher titer of IgG antibodies to *P.gingivalis* than those non-atherosclerotic periodontitis patients. From our research, we can infer *P.gingivalis* infection in AS patients is more serious than that in periodontitis patients without AS. The patients with AS could be infected with *P.gingivalis* with larger quantity or for a longer time than periodontitis patients. The duration and intensity of immune response against *P.gingivalis* may be related with the occurrence of AS.

This present study focused on the relationship between Tregs and *P.gingivalis* infection in atherosclerosis patients. However, the mechanism of how to regulate Tregs response after *P.gingivalis* infection remains unclear. As the limitation of the study, we didn’t analyze the prevalence of the other five genotypes which may also contribute to the progress of AS. Further studies are needed to explain the relationship between *P.gingivalis* infection and the reduction of Treg population.

## Conclusion

Our investigations demonstrate *P.gingivalis* plays an important role in AS, Tregs would be a crucial part in the association of *P.gingivalis* and AS. Genotype II *P.gingivalis* may be a predominate type in this process.

## References

[1] Glass CK, Witztum JL (2001) Atherosclerosis. the road ahead. Cell 104: 503–516. 11239408 10.1016/s0092-8674(01)00238-0

[2] Epstein SE, Zhou YF, Zhu J (1999) Infection and atherosclerosis: emerging mechanistic paradigms. Circulation 100: e20–28. 10421626 10.1161/01.cir.100.4.e20

[3] Kozarov EV, Dorn BR, Shelburne CE, Dunn WA Jr, Progulske-Fox A (2005) Human atherosclerotic plaque contains viable invasive Actinobacillus actinomycetemcomitans and Porphyromonas gingivalis. Arterioscler Thromb Vasc Biol 25: e17–18. 15662025 10.1161/01.ATV.0000155018.67835.1a

[4] Ross R (1999) Atherosclerosis–an inflammatory disease. N Engl J Med 340: 115–126. 9887164 10.1056/NEJM199901143400207

[5] Hansson GK, Jonasson L (2009) The discovery of cellular immunity in the atherosclerotic plaque. Arterioscler Thromb Vasc Biol 29: 1714–1717. 19846836 10.1161/ATVBAHA.108.179713

[6] Tonetti MS (2009) Periodontitis and risk for atherosclerosis: an update on intervention trials. J Clin Periodontol 36 Suppl 1015–19. 19432627 10.1111/j.1600-051X.2009.01417.x

[7] Rutger Persson G, Ohlsson O, Pettersson T, Renvert S (2003) Chronic periodontitis, a significant relationship with acute myocardial infarction. Eur Heart J 24: 2108–2115. 14643271 10.1016/j.ehj.2003.10.007

[8] Miyauchi S, Maekawa T, Aoki Y, Miyazawa H, Tabeta K, et al (2012) Oral infection with Porphyromonas gingivalis and systemic cytokine profile in C57BL/6.KOR-ApoE shl mice. J Periodontal Res 47: 402–408. 22097957 10.1111/j.1600-0765.2011.01441.x

[9] Rodrigues PH, Reyes L, Chadda AS, Belanger M, Wallet SM, et al (2012) Porphyromonas gingivalis strain specific interactions with human coronary artery endothelial cells: a comparative study. PLoS One 7: e52606. 23300720 10.1371/journal.pone.0052606 PMC3530483

[10] Mahendra J, Mahendra L, Kurian VM, Jaishankar K, Mythilli R (2010) 16S rRNA-based detection of oral pathogens in coronary atherosclerotic plaque. Indian J Dent Res 21: 248–252. 20657096 10.4103/0970-9290.66649

[11] Sun W, Wu J, Lin L, Huang Y, Chen Q, et al (2010) Porphyromonas gingivalis stimulates the release of nitric oxide by inducing expression of inducible nitric oxide synthases and inhibiting endothelial nitric oxide synthases. J Periodontal Res 45: 381–388. 20337893 10.1111/j.1600-0765.2009.01249.x

[12] Kebschull M, Demmer RT, Papapanou PN (2010) “Gum bug, leave my heart alone!”–epidemiologic and mechanistic evidence linking periodontal infections and atherosclerosis. J Dent Res 89: 879–902. 20639510 10.1177/0022034510375281 PMC3318075

[13] Grundtman C, Kreutmayer SB, Almanzar G, Wick MC, Wick G (2011) Heat shock protein 60 and immune inflammatory responses in atherosclerosis. Arterioscler Thromb Vasc Biol 31: 960–968. 21508342 10.1161/ATVBAHA.110.217877 PMC3212728

[14] Kleindienst R, Xu Q, Willeit J, Waldenberger FR, Weimann S, et al (1993) Immunology of atherosclerosis. Demonstration of heat shock protein 60 expression and T lymphocytes bearing alpha/beta or gamma/delta receptor in human atherosclerotic lesions. Am J Pathol 142: 1927–1937. 8099471 PMC1886976

[15] Kobayashi R, Kono T, Bolerjack BA, Fukuyama Y, Gilbert RS, et al (2011) Induction of IL-10-producing CD4+ T-cells in chronic periodontitis. J Dent Res 90: 653–658. 21335536 10.1177/0022034510397838 PMC3144111

[16] Sakaguchi S (2005) Naturally arising Foxp3-expressing CD25+CD4+ regulatory T cells in immunological tolerance to self and non-self. Nat Immunol 6: 345–352. 15785760 10.1038/ni1178

[17] O’Neill EJ, Sundstedt A, Mazza G, Nicolson KS, Ponsford M, et al (2004) Natural and induced regulatory T cells. Ann N Y Acad Sci 1029: 180–192. 15681757 10.1196/annals.1309.034

[18] Foks AC, Frodermann V, ter Borg M, Habets KL, Bot I, et al (2011) Differential effects of regulatory T cells on the initiation and regression of atherosclerosis. Atherosclerosis 218: 53–60. 21621777 10.1016/j.atherosclerosis.2011.04.029

[19] Han SF, Liu P, Zhang W, Bu L, Shen M, et al (2007) The opposite-direction modulation of CD4+CD25+ Tregs and T helper 1 cells in acute coronary syndromes. Clin Immunol 124: 90–97. 17512253 10.1016/j.clim.2007.03.546

[20] Ait-Oufella H, Salomon BL, Potteaux S, Robertson AK, Gourdy P, et al (2006) Natural regulatory T cells control the development of atherosclerosis in mice. Nat Med 12: 178–180. 16462800 10.1038/nm1343

[21] Caligiuri G, Groyer E, Khallou-Laschet J, Al Haj Zen A, Sainz J, et al (2005) Reduced immunoregulatory CD31+ T cells in the blood of atherosclerotic mice with plaque thrombosis. Arterioscler Thromb Vasc Biol 25: 1659–1664. 15933243 10.1161/01.ATV.0000172660.24580.b4

[22] Klingenberg R, Gerdes N, Badeau RM, Gistera A, Strodthoff D, et al (2013) Depletion of FOXP3+ regulatory T cells promotes hypercholesterolemia and atherosclerosis. J Clin Invest 123: 1323–1334. 23426179 10.1172/JCI63891 PMC3582120

[23] Ishikawa I, Watanabe H, Horibe M, Izumi Y (1988) Diversity of IgG antibody responses in the patients with various types of periodontitis. Adv Dent Res 2: 334–338. 3271027 10.1177/08959374880020022301

[24] Hayashi F, Okada M, Oda Y, Kojima T, Kozai K (2012) Prevalence of Porphyromonas gingivalis fimA genotypes in Japanese children. J Oral Sci 54: 77–83. 22466890 10.2334/josnusd.54.77

[25] Hansson GK (2005) Inflammation, atherosclerosis, and coronary artery disease. N Engl J Med 352: 1685–1695. 15843671 10.1056/NEJMra043430

[26] Methe H, Brunner S, Wiegand D, Nabauer M, Koglin J, et al (2005) Enhanced T-helper-1 lymphocyte activation patterns in acute coronary syndromes. J Am Coll Cardiol 45: 1939–1945. 15963390 10.1016/j.jacc.2005.03.040

[27] Nilsson J, Wigren M, Shah PK (2009) Regulatory T cells and the control of modified lipoprotein autoimmunity-driven atherosclerosis. Trends Cardiovasc Med 19: 272–276. 20447570 10.1016/j.tcm.2010.02.010

[28] Iwai T (2009) Periodontal bacteremia and various vascular diseases. J Periodontal Res 44: 689–694. 19874452 10.1111/j.1600-0765.2008.01165.x

[29] Chou HH, Yumoto H, Davey M, Takahashi Y, Miyamoto T, et al (2005) Porphyromonas gingivalis fimbria-dependent activation of inflammatory genes in human aortic endothelial cells. Infect Immun 73: 5367–5378. 16113252 10.1128/IAI.73.9.5367-5378.2005 PMC1231143

[30] Hori S, Nomura T, Sakaguchi S (2003) Control of regulatory T cell development by the transcription factor Foxp3. Science 299: 1057–1061. 12522256 10.1126/science.1079490

[31] Kukreja A, Cost G, Marker J, Zhang C, Sun Z, et al (2002) Multiple immuno-regulatory defects in type-1 diabetes. J Clin Invest 109: 131–140. 11781358 10.1172/JCI13605 PMC150819

[32] Crispin JC, Martinez A, Alcocer-Varela J (2003) Quantification of regulatory T cells in patients with systemic lupus erythematosus. J Autoimmun 21: 273–276. 14599852 10.1016/s0896-8411(03)00121-5

[33] Khalaf H, Bengtsson T (2012) Altered T-cell responses by the periodontal pathogen Porphyromonas gingivalis. PLoS One 7: e45192. 22984628 10.1371/journal.pone.0045192 PMC3440346

[34] Kim HJ, Hwang SJ, Kim BK, Jung KC, Chung DH (2006) NKT cells play critical roles in the induction of oral tolerance by inducing regulatory T cells producing IL-10 and transforming growth factor beta, and by clonally deleting antigen-specific T cells. Immunology 118: 101–111. 16630027 10.1111/j.1365-2567.2006.02346.x PMC1782272

[35] Mucida D, Park Y, Kim G, Turovskaya O, Scott I, et al (2007) Reciprocal TH17 and regulatory T cell differentiation mediated by retinoic acid. Science 317: 256–260. 17569825 10.1126/science.1145697

[36] Nakajima T, Ueki-Maruyama K, Oda T, Ohsawa Y, Ito H, et al (2005) Regulatory T-cells infiltrate periodontal disease tissues. J Dent Res 84: 639–643. 15972593 10.1177/154405910508400711

[37] Okui T, Ito H, Honda T, Amanuma R, Yoshie H, et al (2008) Characterization of CD4+ FOXP3+ T-cell clones established from chronic inflammatory lesions. Oral Microbiol Immunol 23: 49–54. 18173798 10.1111/j.1399-302X.2007.00390.x

[38] Amano A (2003) Molecular interaction of Porphyromonas gingivalis with host cells: implication for the microbial pathogenesis of periodontal disease. J Periodontol 74: 90–96. 12593602 10.1902/jop.2003.74.1.90

[39] Nakano K, Inaba H, Nomura R, Nemoto H, Takeuchi H, et al (2008) Distribution of Porphyromonas gingivalis fimA genotypes in cardiovascular specimens from Japanese patients. Oral Microbiol Immunol 23: 170–172. 18279186 10.1111/j.1399-302X.2007.00406.x

[40] Perez-Chaparro PJ, Lafaurie GI, Gracieux P, Meuric V, Tamanai-Shacoori Z, et al (2009) Distribution of Porphyromonas gingivalis fimA genotypes in isolates from subgingival plaque and blood sample during bacteremia. Biomedica 29: 298–306. 20128354

[41] Sugano N, Ikeda K, Oshikawa M, Sawamoto Y, Tanaka H, et al (2004) Differential cytokine induction by two types of Porphyromonas gingivalis. Oral Microbiol Immunol 19: 121–123. 14871353 10.1046/j.0902-0055.2003.00119.x

[42] Kato T, Kawai S, Nakano K, Inaba H, Kuboniwa M, et al (2007) Virulence of Porphyromonas gingivalis is altered by substitution of fimbria gene with different genotype. Cell Microbiol 9: 753–765. 17081195 10.1111/j.1462-5822.2006.00825.x

[43] Mattila KJ, Nieminen MS, Valtonen VV, Rasi VP, Kesaniemi YA, et al (1989) Association between dental health and acute myocardial infarction. BMJ 298: 779–781. 2496855 10.1136/bmj.298.6676.779 PMC1836063

[44] Nalcaci R, Demirer S, Ozturk F, Altan BA, Sokucu O, et al (2012) The relationship of orthodontic treatment need with periodontal status, dental caries, and sociodemographic factors. ScientificWorldJournal 2012: 498012. 23193381 10.1100/2012/498012 PMC3485904

[45] Holmlund A, Hedin M, Pussinen PJ, Lerner UH, Lind L (2011) Porphyromonas gingivalis (Pg) a possible link between impaired oral health and acute myocardial infarction. Int J Cardiol 148: 148–153. 19913930 10.1016/j.ijcard.2009.10.034

[46] Desvarieux M, Demmer RT, Rundek T, Boden-Albala B, Jacobs DR Jr, et al (2005) Periodontal microbiota and carotid intima-media thickness: the Oral Infections and Vascular Disease Epidemiology Study (INVEST). Circulation 111: 576–582. 15699278 10.1161/01.CIR.0000154582.37101.15 PMC2812915

[47] Pussinen PJ, Kononen E, Paju S, Hyvarinen K, Gursoy UK, et al (2011) Periodontal pathogen carriage, rather than periodontitis, determines the serum antibody levels. J Clin Periodontol 38: 405–411. 21362013 10.1111/j.1600-051X.2011.01703.x

[48] Beck JD, Eke P, Heiss G, Madianos P, Couper D, et al (2005) Periodontal disease and coronary heart disease: a reappraisal of the exposure. Circulation 112: 19–24. 15983248 10.1161/CIRCULATIONAHA.104.511998

